# Implementing a protocol to prevent incisional hernia in high-risk patients: a mesh is a powerful tool

**DOI:** 10.1007/s10029-021-02527-0

**Published:** 2021-11-01

**Authors:** J. A. Pereira-Rodríguez, S. Amador-Gil, A. Bravo-Salva, B. Montcusí-Ventura, J. Sancho-Insenser, M. Pera-Román, M. López-Cano

**Affiliations:** 1 Department of General and Digestive Surgery, Hospital, Universitario del Mar. Parc de Salut Mar, Barcelona, Spain; 2 Department of Experimental and Health Sciences, Universitat Pompeu Fabra, Barcelona, Spain; 3 Department of Surgery and Morphological Sciences, Parc de Salut Mar, Hospital del Mar, Universitat Autónoma de Barcelona, Passeig Maritim 25-29, 08003 Barcelona, Spain; 4 Department of General and Digestive Surgery, Hospital Valle de Hebrón, Barcelona, Spain

**Keywords:** Abdominal wall closure, Laparotomy closure, Incisional hernia, Small bites, Short stitch, Prophylactic mesh

## Abstract

**Purpose:**

The small bites (SB) technique for closure of elective midline laparotomies (EMLs) and a prophylactic mesh (PM) in high-risk patients are suggested by the guidelines to prevent incisional hernias (IHs) and fascial dehiscence (FD). Our aim was to implement a protocol combining both the techniques and to analyze its outcomes.

**Methods:**

Prospective data of all EMLs were collected for 2 years. Results were analyzed at 1 month and during follow-up. The incidence of HI and FD was compared by groups (M = Mesh *vs.* S = suture) and by subgroups depending on using SB.

**Results:**

A lower number of FD appeared in the M group (OR 0.0692; 95% CI 0.008–0.56; *P* = 0.01) in 197 operations. After a mean follow-up of 29.23 months (*N* = 163; min. 6 months), with a lower frequency of IH in M group (OR 0.769; 95% CI 0.65–0.91; *P* < 0.0001). (33) The observed differences persisted after a propensity matching score: FD (OR 0.355; 95% CI 0.255–0.494; *P* < 0.0001) and IH (OR 0.394; 95% CI 0.24–0.61; *P* < 0.0001). On comparing suturing techniques by subgroups, both mesh subgroups had better outcomes. PM was the main factor related to the reduction of IH (HR 11.794; 95% CI 4.29–32.39; *P* < 0.0001).

**Conclusion:**

Following the protocol using PM and SB showed a lower rate of FD and HI. A PM is safe and effective for the prevention of both HI and FD after MLE, regardless of the closure technique used.

## Introduction

The beneficial effect for the prevention of incisional hernia (IH) of closing a midline laparotomy with a running suture at a suture length/wound length ratio (SL/WL) of at least 4:1 [1, 2] has been recognized in several randomized controlled trials [3, 4]. The recommendations of societies dedicated to abdominal wall surgery [2] and several comparative studies [3–7] propose combining a high SL/WL ratio with a “small bites” technique (SB) [3, 4], and the use of a prophylactic mesh (PM) [5–7] in high-risk patients. However, both measures have not been widely implemented [8–10], particularly those with high BMI, previous hernia repair, emergency surgery and contaminated/dirty surgery.

There are several reasons for explaining this reluctance, the main ones being that the SB technique has not been satisfactorily studied in high-risk patients, and the potential complications related to PM [8–10].

In a previous study in low-risk patients [11], application of a protocolized closure of the abdominal wall using the SB technique was difficult; however, it was superior in terms of prevention of IH.

We hypothesize that the combination of the SB technique with a PM for closure of the abdominal wall after midline laparotomy reduces the incidence of IH and fascial dehiscence (FD) in high-risk patients.

The main objective of this study is to implement a protocol combining the closure with SB associated with a suprafascial (*onlay*) PM in elective median laparotomy (EML) in high-risk patients and to evaluate its effectiveness for the prevention of complications related to abdominal wall closure.

## Methods

We did a prospective, single-centre, observational study by means The Abdominal Wall Closure Update Hospital Program (PHACPA acronym in Spanish), which is an initiative included in the framework for improving the quality of health and patient care in a University Hospital (IMASQUAL) in Spain.

This program includes changes in the surgical technique of laparotomy closure, unifying the suture material type (polydioxanone 2/0 USP, HR 26 Monoplus^®^, B. Braun. Melsungen, Germany), and using the SB technique and introducing the measurement and systematic documentation of the SL/WL ratio. For that purpose, training actions, which have been previously described, [11] were carried out, and all surgical specialties that perform EML were involved (General and Digestive Surgery, Gynecology, Urology, Vascular). In addition, the use of PM in high-risk patients was emphasised, following updated guidelines [2].

A polyvinylidene (PVDF) mesh (Cicat^®^, Dynamesh, Aachen, Germany) in an *onlay* position, adjusted to the size of the incision with an overlap between 3 and 5 cm, and fixed with a 2/0 polypropylene running suture (Prolene^®^, Johnson and Johnson, NYSE, USA) was used for prophylaxis.

All EMLs in patients with high risk of IH were prospectively included between July 2016 and July 2018. EMLs performed for extraction site or for hand assistance in patients undergoing laparoscopic surgery were also included. The inclusion criteria for the analysis were a minimum of two risk factors for IH [12–14]: age older than 70 years; body mass index (BMI) greater than 30 kg/m^2^; and history of chronic obstructive pulmonary disease (COPD), abdominal aortic aneurysm (AAA), immunosuppression, malnutrition (albumin < 3 g/dL), chronic renal failure (CRF) (creatinine > 1.5 mg/dL), operation for cancer, diabetes mellitus (DM), and smoking. Patients with a previous mesh or hernia present during surgery were excluded.

A common database for all laparotomies (PHACPA study) was designed to collect data of the patient’s characteristics, pathology, operations, surgical wound classification according to Center of Diseases Control (CDC) [15], technique of abdominal wall closure, discharge, complications in the first 30 days classified by the Clavien–Dindo Grade [16] and specifically those of the wound (SSO, SSI, seroma, hematoma, fascial dehiscence and incisional hernia), and follow-up at 1 month, 6 months, 1 year, and thereafter. Data were collected prospectively on a data base designed with FileMaker Pro 15 (Claris International Inc) review of the report and medical records. Clinical follow-up was performed by the surgeon and/or oncologist with physical examination and abdominal CT scan when deemed necessary, data were collected prospectively for the data manager in each appointment. IH was defined according to the description of the EHS [17]: “Any abdominal wall gap with or without a bulge in the area of a postoperative scar perceptible or palpable by clinical examination or imaging”. In patients with persistent purulent discharge, explantation of the mesh would be considered. Patients who presented with FD were operated using abdominal wall closure and suprafascial mesh placement.

**Fig. 1:**
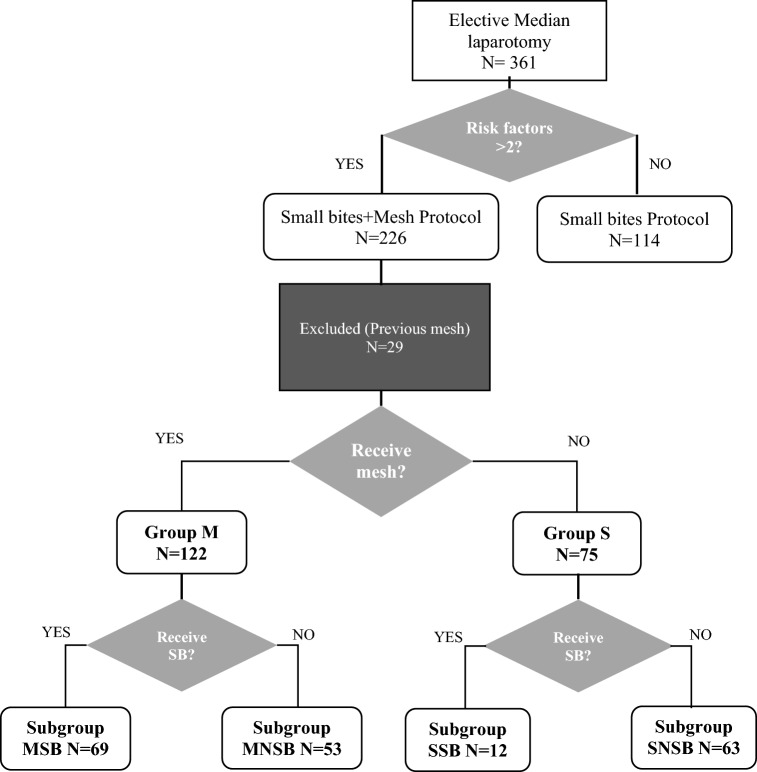
Flowchart

For the analysis, patients were divided into two groups based on the use of PM (M Group) or not (S Group), and they were further divided into subgroups based on the technique of abdominal wall closure. Abdominal wall closure was considered according to the SB technique when the surgical report included the suggested suture material, with the SB technique and the result of the SL/WL ratio. Patients who met these criteria were included in the mesh-small bites (MSB) subgroup if received a PM; the remaining patients were assigned to the suture-small bites (SSB) subgroup. Cases where a different suture material or a different suture gauge was used and/or the calculation of SL/WL ratio was absent were assigned to the mesh-non-small bites (MNSB) subgroup when a PM was used; if not, they were considered in the suture-non-small bites (SNSB) subgroup. The use of SB and PM was at the surgeon’s discretion. The distribution of the groups and subgroups is shown in Fig. 1.

### Statistical analysis

The data were exported to the SPSS 25.0 statistical package (IBM Inc. Rochester, MN, USA). Quantitative variables were expressed as mean ± standard deviation (SD) and qualitative variables as proportions. To analyze the association between qualitative variables, the Chi-squared test or Fisher’s test was used when necessary, as well as Student’s *t* test or the Mann–Whitney test was used for quantitative variables. Normal distribution of the quantitative variables was verified using the Kolmogorov–Smirnov test. Statistical significance was established with *P* < 0.05. A Cox proportional hazards regression model was used to detect the risk factors related to IH.

To reduce the effect of confounding variables, a propensity matching score (PMS) was performed. Scores were estimated by logistic regression analysis, with the treatment strategy group (M *vs.* S) as the dependent variable and age, sex, and previous hernia as independent variables. Matching was performed according to the “nearest neighbor” method using a 0.2-width caliper and at a 2:1 ratio.

The Clinical Research Ethics Committee (CREC number 2016/6543/I) approval was obtained. Patients were informed, and data were processed according to Law 15/1999 on the Protection of Personal Data. The clinical trial protocol was registered with the NCT02658955 code (ClinicalTrials.gov).

## Results

### Demographic data

From July 2016 to July 2018, 226 patients from a University Hospital met the inclusion criteria and underwent surgery for LME among the 82 surgeons comprising part of the Surgical Specialties; 29 patients were excluded for carrying a previous mesh (Fig. 1), and 197 were considered valid for analysis.

Patient characteristics and comparison between the M and S groups are shown in Table 1. These groups showed statistically significant differences in terms of age, sex, and previous hernia repair, which were more frequent in the S group.

**Table 1:** Patient characteristics comparison

	Total *N* = 197	Group M *N* = 122	Group S *N* = 75	*P*
Age, median (IQR)	72.8 (66.9–81.0)	71.9 (66.2–71.9)	75.0 (68.4–81.3)	0.414
Age > 70 years, *N* (%)	124 (62.9)	69 (56.6)	55 (73.3)	0.018
Female sex, *N* (%)	80 (40.6)	59 (48.4)	21 (28.0)	0.005
ASA ≥ III, *N* (%)	111 (56.3)	73 (59.8)	38 (50.7)	0.208
BMI (kg/m^2^), media*N* (IQR)	27.3 (24.2–30.3)	26.5 (23.9–30.0)	27.9 (24.4–30.5)	0.326
BMI > 30 kg/m^2^, *N* (%)	67 (34.9)	38 (31.7)	29 (40.3)	0.226
Smoking, *N* (%)	35 (17.8)	22 (18.0)	13 (17.3)	0.901
DM, *N* (%)	52 (26.4)	32 (26.2)	20 (26.7)	0.946
COPD *N* (%)	51 (25.9)	28 (23.0)	23 (30.7)	0.230
CRF, *N* (%)	20 (10.2)	13 (10.7)	7 (9.3)	0.765
Cancer operation, *N* (%)	168 (85.3)	101 (82.8)	67 (89.3)	0.208
Previous laparotomy, *N* (%)	39 (19.8)	29 (23.8)	10 (13.3)	0.074
Previous hernia, *N* (%)	38 (19.3)	16 (13.1)	22 (29.3)	0.005
Immunosuppression, *N* (%)	16 (8.1)	12 (9.8)	4 (5.3)	0.261
AAA, *N* (%)	6 (3.0)	5 (4.1)	1 (1.3)	0.273

*SD* standard deviation; *IQR* interquartile range; *ASA* American Society of Anesthesiologists; *BMI* body mass index; *DM* diabetes mellitus; *COPD* chronic obstructive pulmonary disease; *CRF* chronic renal failure; *AAA* abdominal aortic aneurysm

### Short-term postoperative comparison

Table 2 presents the data of the operations and their results. Surgery was longer in M group patients, who had a higher frequency of class III and IV wounds. The incision length was shorter in the S group, consistent with a higher proportion of laparotomies for surgical specimen removal (M Group 38 (31.1%) *vs* S Group 44 (58.7%); *P* < 0.0001), although the incision length was recorded in only 15 patients in group S. The proportion of patients with surgical wound classification as type III and IV was higher in group M (15% vs. 5.3%; *P* = 0.038), although the analysis of postoperative complication grade did not show significant differences between the two groups, except for a higher frequency of seromas in patients of the M group (OR 2.686; 95% CI 1.10–6.54; *P* = 0.029). In the postoperative period, only one patient (0.9%) in group M was diagnosed with FD, compared to eight (11.9%) in group S (OR 0.0692; 95% CI 0.008–0.56; *P* = 0.01); of which seven patients were not reported to be using SB (NSB). When comparing the cases after PMS (Table 3), a higher frequency of seromas persisted in the M group, although without statistical significance (OR 1.818; CI 0.86–3.84; *P* = 0.084), and a significant difference in FD was maintained in favor of the M group (OR 0.355; 95% CI 0.255–0.494; *P* < 0.0001).

**Table 2:** Intraoperative characteristics and postoperative complications comparison

	Total *N* = 197	Group M *N* = 122	Group S *N* = 75	*P*
Operative time (minutes), mean (SD)	226.9 (94.3)	238.6 (91.1)	207.8 (96.8)	0.026
Extraction site midline laparotomy, *N* (%)	82 (41.6)	38 (31.1)	44 (58.7)	< 0.0001
SL/WL ratio, median (IQR)^a^	4.75 (4.0–6.1)	4.80 (4.0–6.1)	4.16 (4.0–6.8)	0.783
Length of hospital stay (days), median (IQR)	7.0 (5.0–13.0)	7.0 (5.0–13.0)	6.0 (4.0–13.0)	0.319
Surgical wound classification				
Grade I	61 (30.9)	24 (19.7)	37 (49.3)	
Grade II	114 (57.9)	80 (65.6)	34 (45.3)	
Grade III	21 (10.7)	17 (13.9)	4 (5.3)	
Grade IV	1(0.5)	1 (0.8)	0 (0.0)	
Grade III–IV, *N* (%)	22 (11.3)	18 (15.0)	4 (5.3)	0.038
Specialties				
General and digestive surgery, *N* (%)	125 (63.5)	97 (77.6)	28 (22.4)	
Gynecology, *N* (%)	18 (9.1)	14 (77.8)	4 (22.2)	
Urology, *N* (%)	46 (23.4)	3 (6.5)	43 (93.5)	
Vascular, *N* (%)	8 (4.1)	8 (100)		
Complications grade				
Grade 0, *N* (%)	48 (24.4)	27 (22.1)	21 (28.0)	0.351
Grade I, *N* (%)	37 (18.8)	26 (21.3)	11 (14.7)	
Grade II, *N* (%)	75 (38.1)	49 (40.2)	26 (34.7)	
Grade IIIa, *N* (%)	9 (4.6)	7 (5.7)	2 (2.7)	
Grade IIIb, *N* (%)	22 (11.2)	12 (9.8)	10 (13.3)	
Grade IV, *N* (%)	4 (2.0)	1 (0.8)	3 (4.0)	
Grade V, *N* (%)	2 (1.0)	0 (0.0)	2 (2.7)	0.070
Wound complications				
SSO, *N* (%)	77 (39.1)	50 (41.0)	27 (36.0)	0.486
SSI, *N* (%)	44 (22.3)	27 (22.1)	17 (22.7)	0.930
Superficial SSI, *N* (%)	24 (54.5)	14 (51.9)	10 (58.8)	
Deep SSI, *N* (%)	7 (15.9)	5 (18.5)	2 (11.8)	
Organ space, *N* (%)	13 (29.5)	8 (29.6)	5 (29.4)	
Seroma, *N* (%)	33 (16.8)	26 (21.3)	7 (9.3)	0.029
Hematoma, *N* (%)	12 (6.1)	6 (4.9)	6 (8.0)	0.380
Fascial dehiscence, *N* = 181 (%)	9 (5.0)	1 (0.9)	8 (11.3)	0.002
Subgroups SB, *N* = 77 (%)	2 (2.6)	1 (1.6)	1 (8.3)	0.30
Subgroups NSB, *N* = 104 (%)	7 (6.7)	0 (0.0)	7 (11.9)	0.016

*SD* Standard deviation; *IQR* Interquartile range; *SSO* Surgical site occurrence; *SSI* Surgical site infection

^a^Registered in 15 patients of Group S and in 78 of Group M

**Table 3:** PSM analysis comparison

	Total *N* = 173	Group M *N* = 115	Group S *N* = 58	*P*
Operative time (minutes), mean (SD)	229.5 (92.9)	238.7 (91.7)	212.1 (93.2)	0.811
Extraction site midline laparotomy, *N* (%)	70 (40.5)	34 (58.6)	36 (31.3)	0.001
SL/WL ratio, median (IQR)^a^	4.80 (4.0–6.9)	4.75 (4.0–6.2)	4.80 (4.0–7.6)	0.798
Length of hospital stay (days), median (IQR)	7.0 (5.0–12.0)	7.0 (5.0–12.0)	6.5 (4.0–12.2)	0.593
Surgical wound classification				
Grade I	51 (29.5)	22 (19.1)	29 (50.0)	
Grade II	103 (59.5)	76 (66.1)	27 (46.5)	
Grade III	18 (10.4)	16 (13.9)	2 (3.5)	
Grade IV	1(0.6)	1 (0.9)	0 (0.0)	
Surgical wound classification III–IV, *N* (%)	19 (11.1)	17 (15.0)	2 (3.4)	0.022
Specialties				
General and Digestive Surgery, *N* (%)	114 (65.9)	90 (78.9)	24 (21.1)	
Gynecology, *N* (%)	18 (10.4)	14 (77.8)	4 (22.2)	
Urology, *N* (%)	33 (19.1)	3 (9.1)	30 (90.9)	
Vascular, *N* (%)	8 (4.6)	8 (100.0)	0 (0.0)	
Complications Grade				
Grade 0, *N* (%)	44 (25.4)	26 (22.6)	18 (31.0)	
Grade I, *N* (%)	33 (19.1)	25 (21.7)	8 (13.8)	
Grade II, *N* (%)	63 (36.4)	46 (40.0)	17 (29.3)	
Grade IIIa, *N* (%)	7 (4.0)	5 (4.3)	2 (3.4)	
Grade IIIb, *N* (%)	21 (12.1)	12 (10.4)	9 (15.5)	
Grade IV, *N* (%)	3 (1.7)	1 (0.9)	2 (3.4)	
Grade V, *N* (%)	2 (1.2)	0 (0.0)	2 (3.4)	
Overall complications	129 (74.6)	89 (77.4)	40 (69.0)	0.230
Wound complications				
SSO, *N* (%)	67 (38.7)	45 (39.1)	22 (37.9)	0.878
SSI, *N* (%)	38 (22.0)	24 (20.9)	14 (24.1)	0.624
Superficial SSI, *N* (%)	21 (55.3)	14 58.3)	7 (50.0)	
Deep SSI, *N* (%)	6 (15.8)	4 (16.7)	2 (14.3)	
Organ space, *N* (%)	11 (28.9)	6 (25.0)	5 (35.7)	
Seroma, *N* (%)	30 (17.3)	24 (20.9)	6 (10.3)	0.084
Hematoma, *N* (%)	9 (5.2)	4 (3.5)	5 (8.6)	0.150
Fascial dehiscence, *N* = 158 (%)	9 (5.7)	1 (1.0)	8 (14.5)	< 0.0001
Subgroups SB, *N* = 71 (%)	2 (2.8)	1 (1.7)	1 (10.0)	0.27
Subgroups NSB, *N* = 87 (%)	7 (8.0)	0(0.0)	7 (15.6)	0.012
Incisional hernia, *N* = 141 (%)	20 (14.2)	7 (7.2)	13 (29.5)	< 0.0001
Subgroups SB, *N* = 65 (%)	8 (12.3)	5 (9.1)	3 (37.5)	0.057
Subgroups NSB *N* = 76 (%)	12 (15.8)	2 (4.8)	10 (27.8)	0.009

*SD* standard deviation; *IQR* interquartile range; *ASA* American Society of Anesthesiologists; *BMI* body mass index; *DM* diabetes mellitus; *COPD* chronic obstructive pulmonary disease; *CRF* chronic renal failure; *AAA* abdominal aortic aneurysm

^a^Registered in 12 patients of Group S and in 45 of Group M

### Long-term postoperative comparison

A total of 163 patients completed a mean follow-up of 29.23 months (SD 12.5) (Group M 28.66 *vs*. Group S 25.62; *P* = 0.272), with a statistically significant lower frequency of IH in M group patients (M group 7 (7.2%) *vs* S group 13 (29.5%); OR 0.769; 95% CI 0.65–0.91; *P* < 0.0001). Similar results were obtained by PMS (OR 0.394; 95% CI 0.24–0.61; *P* < 0.0001) (Table 3). No patients of M group presented chronic mesh infection or requires mesh explantation during follow-up.

**Fig. 2:**
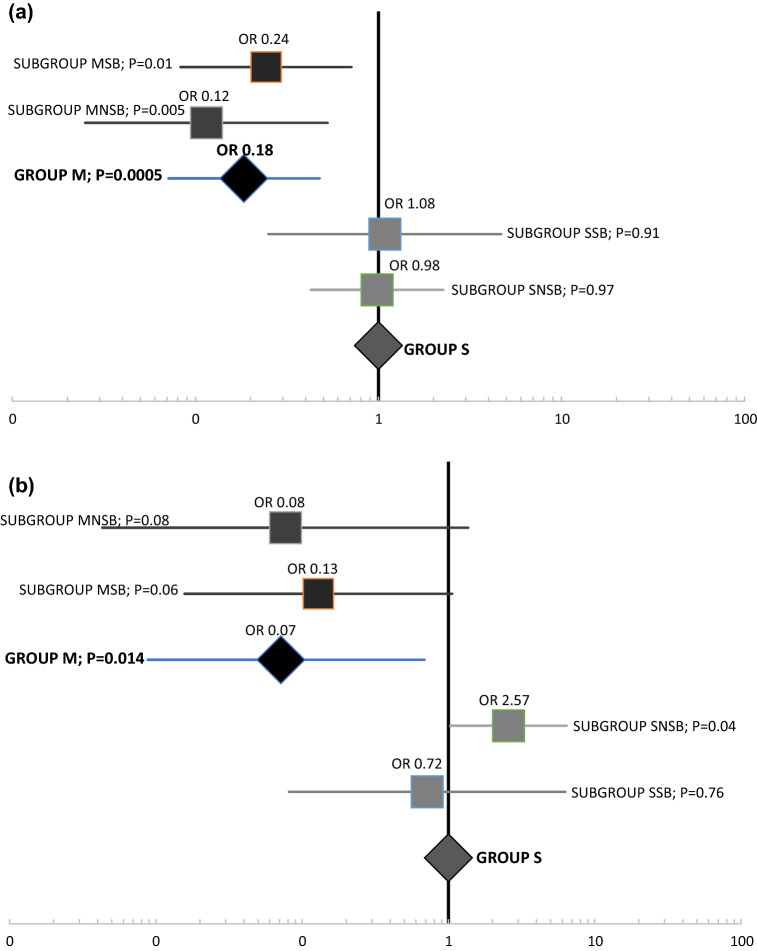
Incisional hernia (**a**) and Fascial dehiscence (**b**) analysis by groups and subgroups. Using Group S as reference

**Fig. 3:**
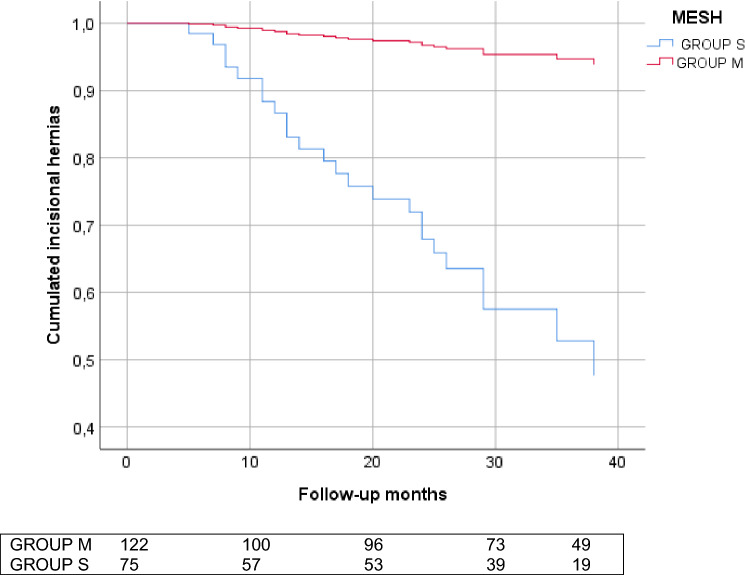
Incisional hernia during follow-up by use of mesh. HR 11.794; CI 4.29–32.39; *P* < 0.0001

Comparing by the subgroups, when a PM was used (Subgroups MSB and MNSB), a more favorable yield was obtained in the incidences of IH and FD regardless of the suturing technique (Table 3 and Fig. 2a, b). Analyzing suturing technique independently, a higher incidence of IH and FD was observed in patients where SB was not performed (MNSB and SNSB) (Table 3). Cox multivariate analysis revealed the use of a PM as the only factor related to prevention of IH (HR 11.794; 95% CI 4.29–32.39; *P* < 0.0001) (Fig. 3).

## Discussion

The results of our study show that adequate fulfillment for closure of EMLs using SB and a PM in high-risk patients correlates with a lower frequency of IH and FD. The main related factor was the use of a PM, independent of the technique for EML closure.

Thus far, studies about EML closure have shown better results when using the SB technique, but they were carried out in non-selected patients [3, 4, 11]. In our study, isolated use of SB, although in a small number of patients, presented a similar frequency of IH than in those treated with NSB, indicating that this group of patients could not benefit as much from this technique as low-risk patients. However, the absence of this data on the operative report was classified as non-compliance with the protocol (NSB), although it could be that it had been correctly applied, a fact that could influence the lack of significance in our sample. We think it is necessary to emphasize to surgeons the importance of measuring and reflecting this data to insure conclusions. Therefore, more research on SB technique in high-risk patients is needed.

For high-risk IH patients, the European Hernia Society (EHS) guidelines [2] suggest the use of a PM, and, in a recent randomized study, the *onlay* position presented greater advantages [18]. Nevertheless, its use has not become widespread for the following reasons: increased costs, possibility of increased complications related to the wound, as well as concerns about legal consequences derived from the use of a prosthesis [9]. As reported previously [7, 18–20], our study shows that using a PM is safe also for contaminated surgery; it is indeed associated with a higher frequency of seromas, but it also implies a clear decrease in the frequency of both IH and FD; thus, in our opinion, the benefit justifies the risk of a minor complication, such as seroma.

FD itself is a serious problem, associated with severe complications and high mortality [21, 23]. Its prevention justifies the use of a PM in these fragile patients who have associated comorbidities to avoid reoperations and, at the same time, prevent a future IH and its impact on the patient’s quality of life and costs. Our data confirm the results of previous studies, in which a PM successfully prevented the appearance of FD [24].

One detail to highlight in our study is that, despite previous teaching work and seminars of the protocol in all participants, a considerable number of surgeons did not fully use it. Our results shows a FD rate of 11.9% and IH rate of 29.5% if the protocol is not followed and this can be improved to 1.6% FD and 9.1% IH observed if the protocol is performed correctly. These data are similar to those reported in previous studies [5–7]. Jairam et al. [18] observed an IH rate in patients with primary suture of 30% versus 13% and 18% observed in patients with onlay and sublay mesh, respectively. Borab et al. [6] described an 85% reduction in the rate of IH with mesh placement and Garcia-Urena et al. [7] documented a rate of 31.5% in the non-mesh control group and 11.3% in the mesh study group.

Only 35% of patients received both PM and SB closure. This result is concerning and highlights the need for encouraging good compliance of protocols and information to surgeons and surgical departments the results of their application. Of particular concern is the low use of SB, and in our study has precluded properly comparing both suturing techniques due to lack of sample. This finding is not exclusive of our study as previously reported [25] only 42% of surgeons followed the EHS guidelines on abdominal wall closure. It is likely that the lack of compliance with SB is related with the lack of measurement of the SL/WL or with lack of confidence on using a 2/0 suture more than a deficiency of knowledge.

It is also striking that in a higher number of procedures, a PM was used (61.9%), which suggests a lower trust by surgeons in the SB technique and a greater penetration as a recommendation for the use of PM in these high-risk patients. It was probably because PM has a longer scientific background [7, 18–27].

In the same line, a higher proportion of incisions in S group patients (58.7%) were related to small/mid-size laparotomies for removal of a specimen or assistance during laparoscopy. Probably, surgeons, when dealing with small incisions, are likely to underestimate the risk of IH and FD. Once analyzed separately, these patients had a higher frequency of FD when not receiving a mesh (M group 1.4% vs. S group 22.2%; *P* < 0.0001), and statistical significance was not reached in IH (M group 9.1% vs. S group 21.1%; *P* = 0.154) probably due to the sample size.

These data confirm that incisions for assistance or specimen extraction in high-risk patients have a similar risk of IH and FD as those with open surgery; therefore, the size of the incision does not seem to be related to IH, as noted previously [27–29].

The main strengths of our study are that it has been carried out prospectively, studying the application in a real setting of a unified technique through learning. The mean follow-up over 2 years ensures there have been no chronic complications related to the use of meshes. Finally, the use of PMS has allowed avoiding biases, which were produced by lack of randomization of patient groups.

There are some weaknesses of our study. The main is related to the decrease in sample size when analyzing subgroups. Also, their characteristics, although it is prospective research, the lack of greater compliance of the protocol makes it difficult to derive definitive conclusions from the comparison of the closure techniques. The suturing technique as well as the use of mesh was at the choice of the surgeon in responsible. During the period of study, all the participating services were informed about postoperative results and the percentage of fulfillment of the protocol every 6 months encouraging them to improve. The specialties of General Surgery, Gynecology and Vascular Surgery were more aware of the use of PM and had better completion of the protocol, while Urology still seems to lack confidence in its use and had worst results. We believe that the correct closure of the abdominal wall should be known and performed equally in all departments that perform LME, given that the wound complications entail a considerable morbimortality for patients, which we believe can be improved.

In conclusion, in patients with risk factors for IHs who are undergoing EML, following the protocol using PM and SB showed a lower rate of FD and HI. A PM is the appears to be a powerful tool for prevention of both IH and FD, regardless the closure technique used.

## Data Availability

The datasets analyzed during the current study are available by request.
